# Tailored Text Messaging Intervention to Improve Self-Care in Patients With Heart Failure (Text4HF): Protocol for a Pilot Randomized Controlled Trial

**DOI:** 10.2196/86667

**Published:** 2026-07-24

**Authors:** Jonathan W Leigh, Susan J Pressler, Ben S Gerber, Mayank Kansal, Mia Cajita, Spyros Kitsiou

**Affiliations:** 1 Department of Biomedical and Health Information Sciences, College of Applied Health Sciences, University of Illinois Chicago, 1919 W. Taylor St., 250 AHSB, MC 530, Chicago, IL, 60612, United States, 1 312-996-7337, 1 312-996-8342; 2 School of Nursing, Indiana University, Indianapolis, IN, United States; 3 University of Massachusetts Chan School of Medicine, Worcester, MA, United States; 4 Department of Medicine, Division of Cardiology, University of Illinois Chicago, Chicago, IL, United States; 5 Department of Biobehavioral Nursing Science, College of Nursing, University of Illinois Chicago, Chicago, IL, United States

**Keywords:** heart failure, mobile health, mHealth, randomized controlled trial, self-care, SMS, text messages

## Abstract

**Background:**

Heart failure (HF) is a major public health problem associated with frequent hospitalizations, high mortality, and substantial health care costs. Self-care is fundamental to improving health outcomes; yet, self-care is commonly poor among patients with HF. SMS text messaging interventions may provide a simple, scalable, and accessible strategy to support HF self-care, particularly among older adults who may face barriers to using more complex digital health technologies. However, the efficacy of text messaging as a standalone intervention for patients with HF remains underexplored.

**Objective:**

This protocol paper describes the rationale and design of a pilot randomized controlled trial examining the feasibility, acceptability, and preliminary efficacy of an individually Tailored Text Messaging Intervention to Improve Self-Care in Adults with HF (Text4HF).

**Methods:**

This study is a single-site, stage I, parallel-group randomized controlled trial. Participants (n=30) are community-dwelling adults aged 50 years or older with stage C HF and suboptimal self-care, defined as a score of 3 or less on at least 2 items of the Self-Care of Heart Failure Index (SCHFI v7.2). Participants are randomized (1:1) to either a 12-week tailored text messaging intervention (Text4HF) plus usual care or usual care alone. Text messages are triggered based on patient responses to validated instruments assessing evidence-based, modifiable behavioral factors of HF self-care. Feasibility (recruitment and retention) and acceptability of the intervention are assessed as key process outcomes. The main exploratory patient-reported outcome is HF self-care (SCHFI v7.2). Other patient-reported outcomes include medication adherence, adherence to a heart-healthy diet, HF knowledge, health-related quality of life, self-efficacy, and health beliefs.

**Results:**

This study was funded in June 2022, and participant recruitment began in September 2024. A total of 26 participants have been enrolled and randomized to the intervention (n=13) and control (n=13) groups. Participants have a mean age of 60 (SD 6.6) years, 46% (12/26) are female, and 73% (19/26) identify as non-Hispanic Black. Half of the participants are individuals with reduced ejection fraction. Study completion is anticipated in June 2026.

**Conclusions:**

This protocol describes an important step toward evaluating a scalable, low-cost text messaging intervention designed to improve self-care in patients with HF. Study findings will provide critical data on feasibility and acceptability to guide a future fully powered efficacy trial of Text4HF.

## Introduction

### Background

Heart failure (HF) is a highly prevalent chronic condition and a leading cause of hospitalization and mortality among middle-aged and older adults (≥50 y) in the United States, contributing to a growing public health burden [1,2]. An estimated 6.7 million Americans have HF, with projections expected to reach almost 9 million by 2030 [2,3]. Epidemiological data indicate that HF disproportionately affects Black people, who experience a higher prevalence (approximately 4.1% compared with 2.8% among White adults), an earlier age of onset (often by a decade), and higher rates of HF-related hospitalization and mortality compared with other racial and ethnic groups [2]. Despite advances in pharmacological and technological therapies, HF-related costs, readmissions, and mortality remain persistently high. Approximately 1 in 4 patients hospitalized for HF are readmitted within 30 days, with an average cost of US $18,000 per readmission [4-6]. These adverse outcomes place substantial strain on health care systems and increase the per capita economic burden of HF.

Evidence suggests that many hospital readmissions may be preventable if patients consistently engage in better HF self-care [7-12]. HF self-care is essential for patients with HF, and improving self-care remains a primary target of multidisciplinary HF management programs. Self-care is a dynamic, patient-centered decision-making process involving routine behaviors and actions that maintain physiological stability (self-care maintenance), facilitate symptom recognition (symptom perception), and guide responses to HF symptoms (self-care management) [13,14]. Clinical guidelines emphasize the central role of patient engagement in HF self-care to improve health outcomes [1,14]. HF self-care comprises several modifiable lifestyle behaviors, including taking medications as prescribed, following a heart-healthy diet, routinely monitoring for HF signs and symptoms (eg, edema, fatigue, and shortness of breath), and engaging regularly in physical activity [1,14]. Systematic reviews of interventions designed to improve HF self-care have demonstrated reductions in hospital readmissions and mortality, as well as improvements in health-related quality of life [9-12].

Despite these benefits, many patients struggle to adhere to guideline-directed self-care behaviors [15-18]. Prior research has identified multiple determinants of poor HF self-care that can be broadly categorized into modifiable behavioral factors and contextual factors. Modifiable behavioral factors include health beliefs, inadequate knowledge about HF and self-care [19-21], low self-efficacy [7,22], forgetfulness [23], and difficulty recognizing symptoms [21,24]. All of these factors are amenable to targeted behavioral interventions. Contextual factors such as socioeconomic and demographic disparities (eg, age, race or ethnicity, and education) as well as clinical characteristics (eg, comorbidities and HF functional status) further influence HF self-care capacity, with racial and ethnic minority populations experiencing disproportionately worse HF outcomes, including higher hospitalization and mortality rates [25]. Although nurse-led patient education and discharge counseling interventions can effectively improve HF self-care, their broader implementation and reach are often limited by reliance on in-person delivery and substantial clinical resources, including staff time and availability. Outside the clinical setting, much of the responsibility for managing HF shifts to community-dwelling patients, who must perform complex self-care behaviors daily. Therefore, there is a critical need to develop and rigorously evaluate low-cost and scalable patient-centered interventions that target evidence-based modifiable behavioral factors of HF self-care, while accounting for broader contextual influences.

Mobile Health (mHealth) technologies including consumer-facing applications and wearables have shown promise for improving HF self-care and reducing hospitalizations [26-30]. Despite this, mHealth adoption and implementation barriers remain common. Patients frequently report challenges related to older age, low digital literacy, fear of new technology, and limited internet access [31]. However, text messaging (TM), or SMS, represents an established and widely used mobile technology that is often overlooked within the broader scope of mHealth tools. Unlike application or device-based interventions, TM requires minimal technical expertise, does not depend on broadband connectivity, and can be delivered at low cost and on a large scale. These characteristics may be particularly relevant for community-dwelling adults with HF, many of whom are older and may face barriers to more complex digital platforms. In a cross-sectional survey of adults with HF (N=100) conducted at a large urban academic health system, 88% reported having an unlimited TM plan and 76% reported using TM regularly [32]. Substantial evidence supports tailoring TM in interventions targeting modifiable self-management behaviors involving chronic conditions such as diabetes [33-35], hypertension [36-38], and obesity [39-41]. In HF, minimal evidence exists of using tailoring in TM interventions [42], but these interventions commonly involve mHealth apps requiring a higher level of technical acumen. Despite promising findings, individually tailored TM interventions targeting modifiable factors influencing HF self-care remain understudied as a standalone intervention. Given the need for low-cost, accessible, and scalable strategies to improve HF self-care, we developed the Tailored Text Messaging Intervention to Improve Self-Care in Patients with HF (Text4HF) study to evaluate the efficacy of a tailored TM intervention for adults with HF.

### Study Objectives

This study aims to determine the feasibility and acceptability of the Text4HF intervention in middle-aged and older adult patients (aged ≥50 y) with HF. It also aims to explore the preliminary efficacy of the Text4HF intervention on HF self-care (primary outcome) and other secondary patient-reported outcomes such as health-related quality of life, medication adherence, heart-healthy diet, and HF knowledge. This trial will provide evidence for the development of a larger trial evaluating the effect of Text4HF on HF self-care.

## Methods

### Study Design

**Figure 1.**
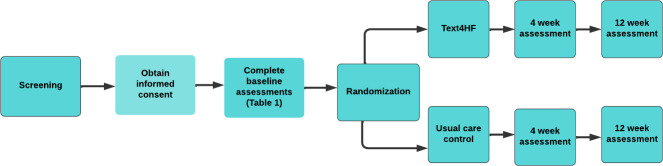
Overview of the study design. Text4HF: Tailored Text Messaging Intervention to Improve Self-Care in Patients with Heart Failure.

The Text4HF study is a stage I, prospective, 2-arm, parallel-group, randomized controlled trial (RCT) in which middle-to-older age (≥50 y) adult patients (n=30) with HF are randomly assigned to either the control group or the Text4HF intervention group for 12 weeks [43,44]. The control group receives usual care, which comprises regular outpatient appointments at the HF clinic for the duration of the study. The intervention group, in addition to usual care, receives a program of individually tailored TM for 12 weeks, targeting modifiable behaviors that influence HF self-care. The follow-up period for the study is 12 weeks, with assessments occurring at baseline, 4 weeks, and 12 weeks. Figure 1 depicts the study flow of participants: (1) eligibility screening, (2) informed consent, (3) baseline assessment, (4) randomization, (5) 4-week assessment, and (6) 12-week assessment. This protocol is reported in accordance with the Spirit guidelines (see appendix 2) [45].

### Population and Setting

This study enrolls patients with stage C HF who demonstrate suboptimal HF self-care. The eligibility criteria are summarized in Textbox 1. Participants are recruited from the University of Illinois Hospital and Health Sciences System (UI Health) in Chicago, Illinois. Potentially eligible patients are identified through the Epic electronic medical record system and are approached by trained research staff, who provide an overview of the study and assess interest in participation. Patients expressing interest undergo additional screening procedures, including the completion of the Self-Care Heart Failure Index (SCHFI) questionnaire [46]. Patients who meet all the eligibility criteria are invited to provide written informed consent prior to study enrollment.

Textbox 1. **Summary of eligibility criteria.**

**Inclusion criteria**

- Aged 50 years or older
- Stage C heart failure (HF; structural heart disease with current or prior HF symptoms [1])
- Actively treated with loop diuretics
- Own a mobile phone with TM plan
- Ability to speak and read English
- Suboptimal HF self-care (Self-Care Heart Failure Index score of 3 or less in at least 2 items of any subscale: Self-Care Maintenance, Symptom Perception, or Self-Management)

**Exclusion criteria**

- Surgical aortic valve replacement, transcatheter aortic valve replacement, ventricular assist device, cardiac resynchronization therapy implantation, and/or heart transplantation scheduled within the next 3 months
- coronary revascularization, and/or cardiac resynchronization therapy implantation within the last 30 days
- Psychosis
- Hospice or end-of-life care
- Advanced renal disease (ie, estimated glomerular filtration rate<25 or hemodialysis)
- Cognitive impairment (ie, dementia and Alzheimer)
- Unable to self-manage (take medication, bathe, use the toilet, and so on)
- Currently living in a nursing home

### Randomization and Allocation Concealment

Following the completion of baseline assessments, enrolled participants are randomized in a 1:1 ratio to either the intervention or control group (n=15 per arm). The randomization sequence is preprogrammed in REDCap (Vanderbilt University) [47] using permuted block randomization stratified by age. Allocation concealment is maintained through a secure, blinded assignment process implemented within the REDCap system. Participants are randomized sequentially in the order in which they complete the baseline assessments. To preserve the integrity of the randomization process, the REDCap system prevents any attempt to change participant assignments following allocation.

### Conceptual Framework

**Figure 2.**
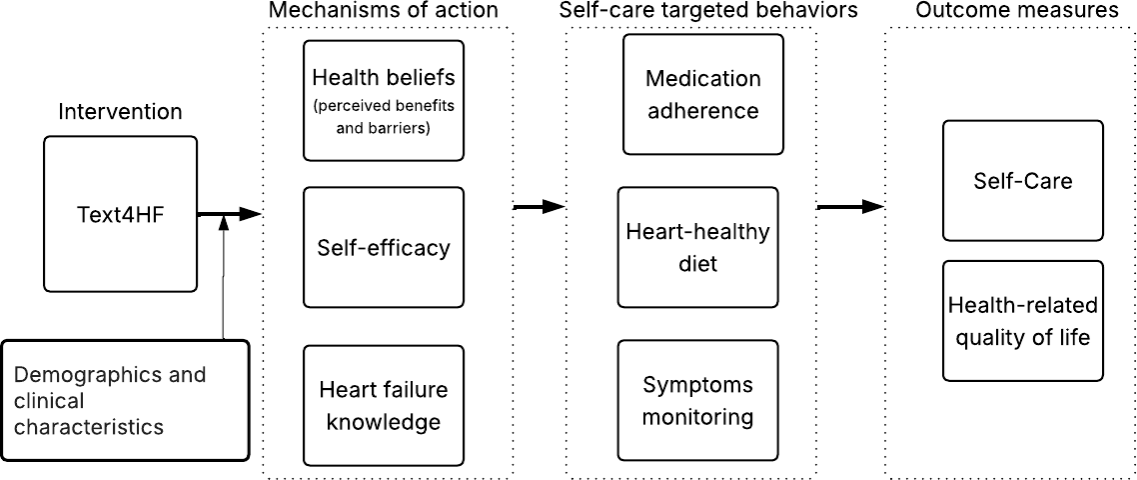
Tailored Text Messaging Intervention to Improve Self-care in Patients with Heart Failure (Text4HF) conceptual framework.

The Text4HF intervention is grounded in the Situation-Specific Theory of Heart Failure Self-Care [13] and the Health Belief Model [48,49]. Guided by these theoretical models, the intervention targets key domains of HF self-care, including self-care maintenance (eg, medication adherence and heart-healthy diet), symptom monitoring (eg, weight changes), and self-care management (eg, seeking care and adjusting diet or loop-diuretics). The TMs are designed to influence the following evidence-based mechanisms of action associated with HF self-care: health beliefs (perceived benefits and barriers regarding HF self-care) [25,50-52], self-efficacy [53-55], and HF knowledge [21,56,57]. Through these mechanisms of action, the intervention aims to improve the ability and confidence of patients with HF to engage in behaviors associated with adequate HF self-care, including medication adherence, dietary adherence, and self-monitoring behaviors. Figure 2 illustrates the conceptual framework underlying the Text4HF intervention, including the proposed mechanisms of action, targeted self-care behaviors, and study outcomes.

### Intervention Development

Details about the development of the Text4HF intervention will be reported elsewhere. Briefly, the Text4HF intervention was developed based on the Heart Messages Program [58] and the iCardia4HF intervention [42,59,60], incorporating the same evidence-based modifiable behavioral determinants of HF self-care (health beliefs, self-efficacy, and HF knowledge), validated assessment instruments, and tailoring strategies, while updating and expanding the TM library and tailoring algorithms to align with current HF clinical guidelines and to more comprehensively address medication adherence, heart-healthy diet, and symptom monitoring.

An interdisciplinary expert advisory team (n=5) with expertise in cardiology, cardiovascular nursing, behavioral TM interventions, and digital health was convened to guide the development of the intervention. The development of the TM library was guided by Kreuter’s [61] step-wise process for tailored health communication. First, the intervention target behaviors and mechanisms of action shown in the conceptual framework were identified. Second, validated instruments to assess these domains and guide message tailoring were selected. Third, we refined existing TMs from the Heart Messages and iCardia4HF intervention studies, and developed new messages informed by HF clinical guidelines [1,62], practical self-care recommendations [14], and patient education resources [63]. Fourth, new rule-based tailoring algorithms were created to link participant assessment responses to personalized TM delivery. The expert advisory team iteratively reviewed the messages to ensure clinical accuracy, behavioral relevance, and consistency with HF management recommendations. To enhance accessibility, all messages were written at approximately an eighth-grade reading level using the Flesch-Kincaid scale, consistent with recommendations for patient education materials [64]. The final TM library consisted of approximately 220 messages linked to the intervention tailoring algorithms. The following section describes the structure, content, and tailoring approach of the Text4HF intervention.

### Text4HF Intervention Description

#### Overview

Participants randomized to the Text4HF intervention group receive 5 individually tailored TMs per week over 12 weeks, for a total of 60 messages, in addition to usual care. These messages are delivered at a time of their preference. The messages are designed to reinforce key HF self-care behaviors, including medication adherence, maintaining a heart-healthy diet, and monitoring symptoms, by addressing common misconceptions and barriers (health beliefs), improving HF knowledge, and increasing self-confidence (Multimedia Appendix 1). Participants complete a baseline assessment and an interim assessment at 4 weeks, coinciding with similar studies involving HF self-care [42,65,66]. Responses from the baseline assessment are used to determine the messages that will be sent for the first 4 weeks, while responses from the 4-week assessment are used to trigger the tailored messages for the remaining 8 weeks of the intervention.

#### Tailoring Approach

Triggering and tailoring of the TMs are guided by participants’ responses to five validated instruments: (1) Beliefs About Medication Compliance Scale (BMCS); (2) Beliefs About Diet Compliance Scale (BDCS); (3) Beliefs About Self-Monitoring Scale (BSMS) [67,68]; (4) Adherence to Refills and Medications Scale (ARMS) [69]; and (5) Scale for Dietary Behavior in Heart Failure (SDBHF) [70]. Tailoring is conducted using predetermined rule-based algorithms and scoring thresholds derived from the response scales of each instrument (Multimedia Appendix 1). For example, in Table 1, questions on the Health Beliefs Scales (BMCS, BDCS, and BSMS) are divided into perceived benefit and barrier questions. Each question is scored on a 5-point Likert scale ranging from 1 (“strongly disagree”) to 5 (“strongly agree”). Participants who respond with a score of 3 or more on a barrier question or 3 or less on a benefit question receive messages tailored to that specific barrier or benefit item (Table 1). If a participant scores more than 3 on a benefit question or less than 3 on a barrier question, then a message related to that item is not sent because it is presumed that the patient already understands the barriers or benefits identified in that question. The ARMS and SDBHF use a 4-point Likert scale. A cutoff of 2 or higher for regularly coded items and scores of 3 or less for reverse-coded items was used.

**Table 1.** Tailored text message (TM) examples.

Assessment scale	Sample item	Algorithm		TM
		TM	No TM	
Health beliefs				
BMCS**^a^**	Taking water pills makes me wake up at night to go to the bathroom	Assessment scores of 3 (“undecided”), 4 (“agree”), or 5 (“strongly agree”)	Assessment scores of 1 (“strongly disagree”) and 2 (“disagree”)	People who take water pills often wake up at night to go to the bathroom. Check with your doctor or nurse to see if you can take your pills earlier as it is recommended to take water pills at least 6 hours before bedtime.
BDCS**^b^**	Food does not taste good on the low salt diet	Assessment scores of 3 (“undecided”), 4 (“agree”), or 5 (“strongly agree”)	Assessment scores of 1 (”strongly disagree”) and 2 (“disagree”)	Avoid herb or spice mixtures that contain salt or sodium. Use lemon, orange, pineapple juice, or fresh ground pepper for flavor.
BMCS**^c^**	Weighing myself everyday takes too much time	Assessment scores of 3 (“undecided”), 4 (“agree”), or 5 (“strongly agree”)	Assessment scores of 1 (“strongly disagree”) and 2 (“disagree”)	Set aside a few minutes every day to weigh yourself. It helps you find out if there is too much fluid in your body.
Medication adherence				
ARMS**^d^**	How often do you run out of medicine?	Assessment scores of 3 (“undecided”), 4 (“agree”), or 5 (“strongly agree”)	Assessment score of 1 (“none of the time”)	Take your medication at the same time as an activity you do every day such as cooking, brushing your teeth, or watching your favorite TV show.
Heart-healthy diet				
SDBHF**^e^**	I add the soups I consume, the meals I eat and the fruits (vegetables and fruits with high water content) to the daily liquid consumption account.	Assessment scores of 1 (“never”), 2 (“sometimes”), or 3 (“frequently”)	Assessment score of 4 (“always”)	Fill a container with 2 liters (2000 cc) of water (or whatever your limit is for the day). Each time you drink/eat fluid, take the same amount of fluid out of the container. When the container is empty, you will know you have had your fluid limit for the day.

a BMCS: Beliefs About Medication Compliance Scale.

b BDCS: Beliefs About Diet Compliance Scale.

c BSMS: Beliefs About Self-Monitoring Scale.

d ARMS: Adherence to Refills and Medication Scale.

e SDBHF: Scale of Diet Behavior in Heart Failure.

#### Text Message Prioritization

In cases where participants trigger more TMs than the total intervention dose of 60 messages over the 12-week intervention period, the tailoring algorithm is designed to prioritize TMs based on the degree of participant response to each Likert-scale assessment item. The most extreme responses indicative of greater self-care barriers or support needs are assigned higher priority levels, whereas less extreme responses are assigned medium or low priority levels. This prioritization approach is intended to ensure that participants receive TMs most relevant to their individual self-care needs. Examples of the prioritization process are provided in Multimedia Appendix 1.

#### Text Message Delivery

The TMs are programmed and sent via the iCardia (Kitsiou) [71] platform by research staff within 24 hours of the completion of the assessments. Participants indicate a convenient time at which they would like to receive the messages 5 days per week. One sample item from each assessment and one example TM are shown in Table 1.

#### Patient Safety Considerations

The Text4HF intervention is not intended to replace clinical care or function as a telehealth intervention. Participants are instructed to contact their health care provider or seek emergency medical care if they experience worsening symptoms or require urgent medical attention. Patients with HF may opt out of the TM at any time by texting "Stop," which is explained during the informed consent process.

### Control Group (Usual Care) Description

Participants allocated to the control group receive standard outpatient care through the UI Health Heart Failure Clinic. Usual care includes routine follow-up visits with the HF care team, including HF cardiologists, advanced practice nurses, and clinical pharmacists. Follow-up visits typically occur every 3 to 6 months, depending on the condition of the patient with HF. During these visits, advanced practice providers assess and titrate current HF guideline-directed medications, care providers perform clinical assessments, optimize guideline-directed medical therapy, coordinate referrals, and follow-up care (ie, primary care and cardiac rehabilitation), provide posthospitalization management, and engage in ongoing treatment planning. Participants also receive patient-centered HF self-care education based on materials developed by the American Heart Association’s Get With The Guidelines program [62].

### Study Measures and Data Collection

#### Overview

Study measures are presented by the study aim in Table 2, which includes the outcome, the instrument or data collection tool, a description of each assessment, and the schedule of assessments.

**Table 2.** Measures, instruments, and assessment schedule.

Outcomes	Instruments or data collection tool	Description	Schedule
Feasibility and acceptability measures (aim 1)			
Screening	REDCap-generated reports	Number of patients screened for eligibility, proportion of screened patients who are eligible (eligibility rate), reasons for exclusion, and number of patients screened to identify one eligible participant.	Throughout the study
Recruitment	REDCap-generated reports	Number of participants invited to participate in the study. Number of participants who declined participation. Number of participants who signed the informed consent form. Percentage of eligible patients who consent (enrollment rate). Percentage of consented patients who complete baseline assessment and are randomized (randomization rate). The time required to achieve the target sample size (expressed in months). The number of participants enrolled per month (accrual rate).	Throughout the study
Retention	REDCap-generated reports	Proportion of participants who: complete the study (completion rate), voluntarily withdraw (withdrawal rate), and who are LTFU^a^ rate.	Throughout the study
Intervention fidelity	iCardia system report; and TAM^b^ [72] Survey (1-item self-report of messages read)	Percentage of TM^c^ successfully delivered. Participant postintervention self-report of overall TM read (none, some, about half, nearly all, or all).	Throughout the study
Assessment completion	REDCap-generated reports	Number of patients who completed 4-week and 12-week assessments.	Throughout the study
Acceptance and satisfaction	TAM^b^ [72] Survey (self-report)	23-item questionnaire assessing perceived usefulness, satisfaction, and acceptance of the TM intervention	12 weeks
Patient-reported outcomes (aim 2)			
Self-care	SCHFI^d^ v7.2 [13,73] (self-report)	Self-reported self-care assessed with 3 subscales: Self-Care Maintenance, Symptom Perception, and Self-Care Management. Subscale scores range from 0 to 100. A score of ≥70 indicates adequate HF^e^ self-care.	Baseline, 4 weeks, and 12 weeks
Medication adherence	ARMS^f^ [69] (self-report)	A 12-item, 4-point Likert medication adherence scale. Lower total scores indicate better adherence.	Baseline, 4 weeks, and 12 weeks
Diet adherence	SDBHF^g^ [70] (self-report)	A 12-item, 4-point Likert diet adherence scale. A total score less than 46 indicates lack of adherence to the diet guidelines.	Baseline, 4 weeks, and 12 weeks
Health-related quality of life	MLHFQ^h^ [74,75] (self-report)	A 21-item, 5-point Likert scale assessing HF symptoms and impact on quality of life. Higher scores indicate lower quality of life.	Baseline, 4 weeks, 12 weeks
Self-care self-efficacy	SCSE^i^ SCHFI subscale [13,73] (self-report)	Measured with the SCHFI Self-Care Self-Efficacy Subscale (10 items). Scores range from 0 to 100. A score of ≥70 indicates adequate self-care efficacy.	Baseline, 4 weeks, and 12 weeks
HF knowledge	AHFKT^j^ [76] (self-report)	Assessed with the AHFKT, including 30-item multiple choice and yes or no questions. The higher the score, the greater the HF knowledge.	Baseline, 4 weeks, and 12 weeks
Health beliefs	BDCS^k^, BMCS^l^, and BSMS^m^ [67,68] (self-report)	Self-reported HF health beliefs (benefits and barriers) assessed with 3 subscales (medication, low salt diet, and symptoms monitoring). A total of 42 items and a 5-point Likert scale questionnaires.	Baseline, 4 weeks, and 12 weeks
Demographics and clinical characteristics			
Adverse events	EHR^n^	Hospitalizations and emergency department visits.	Baseline, 4 weeks, and 12 weeks
Clinical HF characteristics	EHR^n^	NYHA^o^ class and left ventricular ejection fraction, medications, HF etiology, and comorbidities.	Baseline
Comorbidities	CCI^p^ [77-79] (EHR)	The CCI is a calculator that derives a score to predict 10-year mortality rates.	Baseline
Cognition	EHR^n^	Cognitive status abstracted from the EHR at screening.	Baseline
Demographics	Self-report	Age, employment, race or ethnicity, education, insurance status.	Baseline
Depression	PHQ-8^q^ [80,81] (self-report)	PHQ-8 is a validated questionnaire to assess depressive symptoms.	Baseline

a LTFU: lost-to-follow-up.

b TAM: Technology Acceptance Model Questionnaire.

c TM: text messaging.

d SCHFI: Self-Care Heart Failure Index.

e HF: heart failure.

f ARMS: Adherence to Medication Scale.

g SDBHF: Scale for Diet Behavior in Heart Failure.

h MLHFQ: Minnesota Living with Heart Failure Questionnaire.

i SCSE: Self-Care Self-Efficacy.

j AHFKT: Atlanta Heart Failure Knowledge Test.

k BDCS: Beliefs About Diet Compliance Scale.

l BMCS: Beliefs About Medication Compliance Scale.

m BSMS: Beliefs About Self-Monitoring Scale.

n EHR: electronic health record.

o NYHA: New York Heart Association.

p CCI: Charlson Comorbidity Index.

q PHQ-8: Patient Health Questionnaire–8.

#### Feasibility and Acceptability (Aim 1)

Feasibility will be assessed across 4 domains: screening, recruitment, retention, and intervention.

Screening feasibility refers to the practicality and efficiency of identifying eligible participants from the target population. Screening feasibility measures in our study include the total number of patients screened for eligibility, the proportion of screened patients who are eligible (eligibility rate), reasons for exclusion, and the number of individuals who must be screened to identify one eligible patient.

Recruitment feasibility evaluates the ability of the study team to successfully enroll eligible participants into the trial within the planned timeframe, using the planned recruitment methods. Recruitment feasibility measures in the Text4HF trial include the number of potential participants invited to participate in the study, those who declined participation, and those who provided informed consent; the percentage of eligible patients who consented (enrollment rate); the percentage of consented patients who provided consent, completed the baseline assessment, and were randomized (randomization rate); the time required to achieve the target sample size (expressed in months); and the number of participants enrolled per month (accrual rate).

Retention feasibility refers to the ability to keep enrolled participants in the study until the planned outcome assessments are completed. Retention feasibility is assessed in our trial by the proportion of randomized participants who remain in the study and complete the final follow-up assessment (completion rate). Additional measures of retention include the proportion of participants who voluntarily withdraw from the study (withdrawal rate) and the proportion of participants who are lost to follow-up (LTFU rate).

Intervention feasibility refers to the extent to which the TM intervention is delivered and received as intended by the study protocol. Intervention feasibility is assessed using 2 measures. The first measure is the percentage of TM successfully delivered, as determined by reports generated by the intervention delivery system (iCardia). The second measure is a self-report administered at the end of the intervention, which asks participants approximately how many of the study TM messages they received and read (none, some, about half, nearly all, or all).

Acceptability is assessed with an exit survey completed by all participants in the intervention group. The exit survey was developed solely for the purpose of this study and was adapted from the Technology Acceptance Model [82]. We designed a 23-question self-reported survey involving 19 Likert scale questions and 4 open-ended free text questions. The questions gather information such as perceived usefulness, satisfaction, and intention to continue receiving TM. The open-ended questions focus on features of the TM intervention that participants liked or disliked.

#### Patient-Reported Health Outcome Measures (Aim 2)

The primary outcome of interest is HF self-care, assessed with the SCHFI version 7.2 [46] at baseline, 4 weeks, and 12 weeks. The SCHFI (29 items) has 3 subscales: Self-Care Maintenance (10 items), Symptom Perception (11 items), and Self-Care Management (8 items). Standardized scores in the SCHFI range from 0 to 100. A total score of 70 or more indicates adequate self-care [13,73].

Secondary patient-reported health outcomes include medication and refill adherence (ARMS), heart-healthy diet adherence (SDBHF), HF knowledge (Atlanta Heart Failure Knowledge Test [AHFKT]), health-related quality of life (Minnesota Living with Heart Failure Questionnaire [MLHFQ]), self-efficacy (Self-Care Self-Efficacy [SCSE]), and health beliefs (BDCS, BMCS, and BSMS) at baseline, 4 weeks, and 12 weeks.

As part of the safety protocol, the study team will review the EHR and record adverse events, serious adverse events, and unanticipated problems, including unplanned hospitalizations, emergency department visits, and mortality or death events for all enrolled participants.

#### Demographics and Clinical Characteristics

Data collected will be a combination of self-reported information and data abstracted from electronic medical records. Demographic information includes the following (eg, name, date of birth, employment status, insurance, ethnicity, race, and education). Contact information includes address, telephone number, email address, HF clinical characteristics, such as New York Heart Association classification and left ventricular ejection fraction, will also be collected, along with medical history, past medical conditions and illnesses, medications, and hospitalizations. The Patient Health Questionnaire [80,81] (8 items) will be used to assess depression status. Comorbid conditions will be collected and scored using the Charlson Comorbidity Index [77-79] (which includes 19 comorbidities).

### Statistical Analysis Plan

#### Sample Size Justification for a Pilot RCT

Given the pilot nature of this RCT, the sample size was not based on a formal-efficacy-powered calculation. Instead, it was determined based on feasibility considerations and estimation of preliminary outcome variability. The main objectives of the study are to evaluate the feasibility, acceptability, and preliminary effects of the Text4HF intervention and to generate estimates of variability and effect sizes to inform a future, fully powered trial. The primary patient-reported outcome is HF self-care, measured using the SCHFI, which yields continuous scale scores and can be used to estimate between-group differences, variability, and effect sizes for a future definitive trial. Consistent with recommendations for pilot RCTs involving continuous behavioral outcomes [83,84], we determined that we need to enroll 30 participants and randomize them equally between study arms (n=15 per group) to inform a 90% powered trial with an expected standardized effect size in the medium range (0.3≤*d*<.7) [84]. This sample size is appropriate for estimating feasibility parameters, retention, intervention acceptability, and preliminary changes in SCHFI scores, while also providing estimates of SCHFI variability and effect size needed to design a future fully powered trial. Assuming up to 20% attrition over 12 weeks, approximately 24 participants are expected to complete the 12-week follow-up assessment. The target sample size also balances methodological recommendations for pilot RCTs with the available study resources, timeline, and anticipated recruitment capacity.

#### Analysis Plan

The primary analyses comparing the intervention and usual care groups will follow the intention-to-treat principle, whereby participants are analyzed according to the group to which they were originally randomized, regardless of intervention adherence or exposure.

Standard descriptive statistics will be used to describe recruitment and retention rates, participant characteristics, and intervention acceptability. Chi-square tests and *t* tests will be used to examine differences between the groups in exploratory patient-reported outcomes (Table 2). Cohen *d* effect sizes will be used to evaluate the preliminary effect of the text messages on HF self-care measures as assessed by the SCHFI (Table 2). The significance level will be set at less than .05 for all analyses.

### Data Collection and Management Procedures

This study uses three systems for data collection and intervention delivery: (1) REDCap, (2) Epic, and (3) iCardia. The REDCap database is used for documenting screening, enrollment, and storing intervention-related data. The Epic UI Health electronic health records system is used to abstract clinical data and document informed consent from enrolled participants. iCardia [71] is a secure, internet-based application that was developed at the university and is used to program and send TM. All data are verified, cleaned, and analyzed using SPSS (version 30.0; IBM Corp).

### Ethical Considerations

The study is conducted in accordance with the Declaration of Helsinki, the International Council for Harmonization (Good Clinical Practice), and the laws and local regulations applicable in the United States. The study is approved by the Institutional Review Board (IRB protocol #2022‐0704) and is registered at ClinicalTrials.gov. Appropriate IRB waivers for electronic medical record review and research participant recruitment were granted by the University of Illinois Chicago IRB. Trained research assistants obtain informed consent using the REDCap [47] eConsent Framework once eligibility and patient interest are confirmed. Trained research assistants obtain informed consent via the REDCap [47] electronic consenting platform once eligibility and patient interest are confirmed. As part of our safety monitoring protocol, we record all hospital admissions, emergency room visits, and deaths. These events are documented in the study record by a study team member. Participants receive US $30 per study visit as compensation for their time and effort for a total of US $60 if both assessments are completed.

To protect the privacy of study participants and to maintain the confidentiality of the data, all datasets created in this study are stored electronically in secure, password-protected, and encrypted databases. All human participants are assigned a Subject ID code in REDCap, which is used during data collection throughout the study. All computerized and database systems used in this study for data collection and analysis are password-protected and encrypted, and will be accessible only to the University of Illinois Chicago key research personnel.

### Dissemination Plan

The findings from this trial will be disseminated at a conference as a poster or oral presentation, as well as through a manuscript that will be published in an open access, peer-reviewed journal.

## Results

This trial is funded by the Midwest Roybal Center for Health Promotion and Translation in June 2022 and received approval from the University of Illinois Chicago IRB. Start-up activities were completed by August 2024, including obtaining IRB approval, creating data collection instruments, developing the TM library, and testing the TM algorithm. Recruitment began in September 2024. Overall, 26 participants have enrolled in the study and have been randomized to the intervention group (n=13) and the control group (n=13). The mean age of participants is 60 (SD 6.62) years, and 46% (12/26) are female. Regarding self-identified race, 73% (19/26) are non-Hispanic Black. The majority of the patients are clinically evaluated as NYHA Class II (19/26, 73%), with 50% (13/26) diagnosed with reduced ejection fraction. The estimated completion date of the study is June 30, 2026. Results of the study are expected to be published later this year or in 2027.

## Discussion

### Anticipated Findings

Although TM has demonstrated effectiveness in self-management interventions, such as diabetes, hypertension, and obesity, evidence as a standalone study in individuals with HF is limited. Many of these studies on self-management are deployed in conjunction with remote patient monitoring. The Text4HF trial aims to address an important gap in the literature by providing preliminary evidence on the feasibility and efficacy of individually tailored text messages as a standalone intervention for improving self-care in middle-aged and older adult patients with HF. Given the potential for scalability, reach, and low cost of TM, this approach could evolve into a paradigm shift where TM could be deployed in an HF clinic. The study is not powered to establish efficacy; rather, it is intended to generate estimates of effect size and variability that will inform the design of a future fully powered RCT. We anticipate the study will demonstrate feasibility with recruitment and retention rates greater than 80%. Acceptability will be demonstrated with high rates of patient satisfaction and preliminary improvements in HF self-care compared with usual care.

### Comparison With Prior Work

In 2019, Chen et al [85] conducted a large (n=767) RCT in China that provided one-way, Heart Failure Society of America-based, educational TMs for a month to patients recently discharged from a HF-related hospitalization [40]. While the Chen et al [85] study illustrated that this approach is feasible and cost-saving, the messages were not individually tailored. iCardia4HF [42] was a complex, multicomponent mHealth intervention that included several Bluetooth-enabled devices and mobile apps, along with individually tailored TMs. The TMs created in iCardia4HF were based on the Heart Messages program [43] and focused on 3 critical domains: adherence to loop diuretics, a low-sodium diet, and self-monitoring of weight and edema. However, there is a need to further enhance and expand the existing messages. The messages should holistically address unhealthy lifestyle behaviors and ensure that the program aligns with the latest clinical guidelines for patients with HF. Additionally, it is equally important to assess the independent effect of individually tailored TMs to improve HF self-care as a standalone intervention.

Cornelius et al [86] targeted patients with HF in a small (n=30) randomized controlled pilot study involving urban patients who were provided with 2-way TM reminders to improve depression and anxiety [87]. However, TMs were used to deliver questions for the reinforcement of cognitive behavioral therapy sessions and did not include self-care related content.

The MESSAGE-HF was a large (n=699) RCT involving TM in conjunction with nurse-led telemonitoring [88]. The dose (frequency) of messages was adjusted based on the telemonitoring feedback. However, the authors reported no clinically meaningful changes in clinical laboratory levels, mortality, or hospitalizations [88].

### Strengths

TM is widely used and has the potential to meet the needs of patients with varying levels of technology literacy. Prior studies of TM interventions have used a one-size-fits-all approach where all patients receive a fixed dose of messages regardless of their health beliefs, knowledge of their condition, or experience with self-care. Personalization or tailoring of the messages is usually centered on including participants’ names or using other surface-level approaches. Only one other study, iCardia4HF, has used this methodology to improve self-care behavior, but it combined TM with multiple mHealth apps and connected health devices, making it difficult to assess the effects of TM as a standalone intervention [59].

The patient population demographics in the Text4HF study are approximately 80% Black or African American, 10% Hispanic or Latinx, and 10% White. This study will provide valuable data on addressing gaps in research with respect to enrolling older, low-income Black or African American patients with HF who carry the highest risk of hospitalization, morbidity, and mortality [17]. Additionally, women have traditionally been underrepresented in HF research [89-92], and this study is well-positioned to address these gaps.

The Text4HF study has the potential to address the needs of patients with HF through a simple, low-technological, yet sophisticated intervention. Improvements in patient HF self-care among patients using existing technology represent an exciting way to reach patients in the community with a low-cost solution.

### Limitations

This study has a few limitations. First, this is a single-site pilot study recruiting participants from an urban academic hospital setting. The population consists predominantly of patients from racial and ethnic minority groups and may not be generalizable to other demographic populations. Second, perceptions may play a role in the results because they are based on patient-reported outcomes (self-care), and the lack of blinding may contribute to performance bias. Therefore, since this is a stage I behavior change intervention, the results on the preliminary effect of the intervention on exploratory patient-reported outcomes from this study should be interpreted cautiously.

### Future Directions

The Text4HF intervention will provide data on feasibility, acceptability, and preliminary efficacy to refine the study for a larger clinical trial in the future. Future work involving TM in HF may provide the basis for deploying this methodology into clinical practice.

## Supplementary material

10.2196/86667 Multimedia Appendix 1 Text message triggering, prioritization, and sample messages.

10.2196/86667 Checklist 1 SPIRIT checklist.

## Abbreviations

AHFKT: Atlanta Heart Failure Knowledge Test
ARMS: Adherence to Refills and Medications Scale
BDCS: Beliefs About Diet Compliance Scale
BMCS: Beliefs About Medication Compliance Scale
BSMS: Beliefs About Self-Monitoring Scale
HF: heart failure
IRB: Institutional Review Board
LTFU: lost-to-follow-up
mHealth: mobile health
MLHFQ: Minnesota Living with Heart Failure Questionnaire
RCT: randomized controlled trial
SCHFI: Self-Care Heart Failure Index
SCSE: Self-Care Self-Efficacy
SDBHF: Scale for Dietary Behavior in Heart Failure
Text4HF: Tailored Text Messaging Intervention to Improve Self-Care in Patients with Heart Failure
TM: text messaging
UI Health: University of Illinois Hospital and Health Sciences System
