# Supplementary material for: Tailored Text Messaging Intervention to Improve Self-Care in Patients With Heart Failure (Text4HF): Protocol for a Pilot Randomized Controlled Trial
Source: JMIR Res Protoc. 2026 Jul 24;15:e86667. doi: 10.2196/86667 (PMC13399410; doi:10.2196/86667)
Supplement: Multimedia Appendix 1 [file resprot-v15-e86667-s001.docx]

**Multimedia Appendix**

We expected participants to trigger more messages than the total dose (60 TM) over the 12-week intervention. Therefore, considerations were made during the algorithm design process to prioritize the TM based on the degree of participant response to each of the Likert-style assessment questions. The most extreme Likert responses from each of the questions on the intervention target assessment tools are considered high priority. The next set of responses are medium priority. The last possible responses are low priority. If two questions trigger TMs considered high priority those messages are sent until they reach the threshold of 3 TM per assessment question, which is the total number of messages created per item. Once the threshold is reached, then the medium priority and low priority messages are sent. If no high priority messages are triggered for an instrument, then the medium are sent first until the threshold is met followed by the low priority messages. This way, the most extreme responses are prioritized and tailored to each participant. Examples of the prioritization are found below for the health belief scales (i.e. BMCS, BDCS, and BSMS), ARMS, and SDBHF.

***Triggering:*** The HBS (i.e., BMCS, BDCS, and BSMS) scales triggering the TM by responses to each item depending on the type of question (i.e. benefit, barrier).

| HBS Scales TM Triggering (5-point Likert Scale) | | | | | |
| --- | --- | --- | --- | --- | --- |
|  | Strongly Agree | Disagree | Undecided | Agree | Strongly Agree |
| Benefit Question | 1[TM] | 2[TM] | 3[TM] | 4 | 5 |
| Barrier Question | 1 | 2 | 3[TM] | 4[TM] | 5[TM] |

**Table 1: HBS TM Triggering based on participant responses**

***Prioritization****:* Scores of 1 (Benefits) and 5 (Barriers) are the highest priority response, 2 (Benefits) and 4 (Barriers) are the medium priority response, 3 (Benefits) and 3 (Barriers) are the lowest priority. Responses of 4 or 5 (Benefits) or 1 or 2 (Barriers) do not receive TM.TM are prioritized by response to each item 1 (regular coded) and 5(reverse coded) are the highest priority response, 2 (regular coded) and 4 (reverse coded) are the medium priority response, 3 (regularly) and 3 (reverse coded) are the lowest priority.


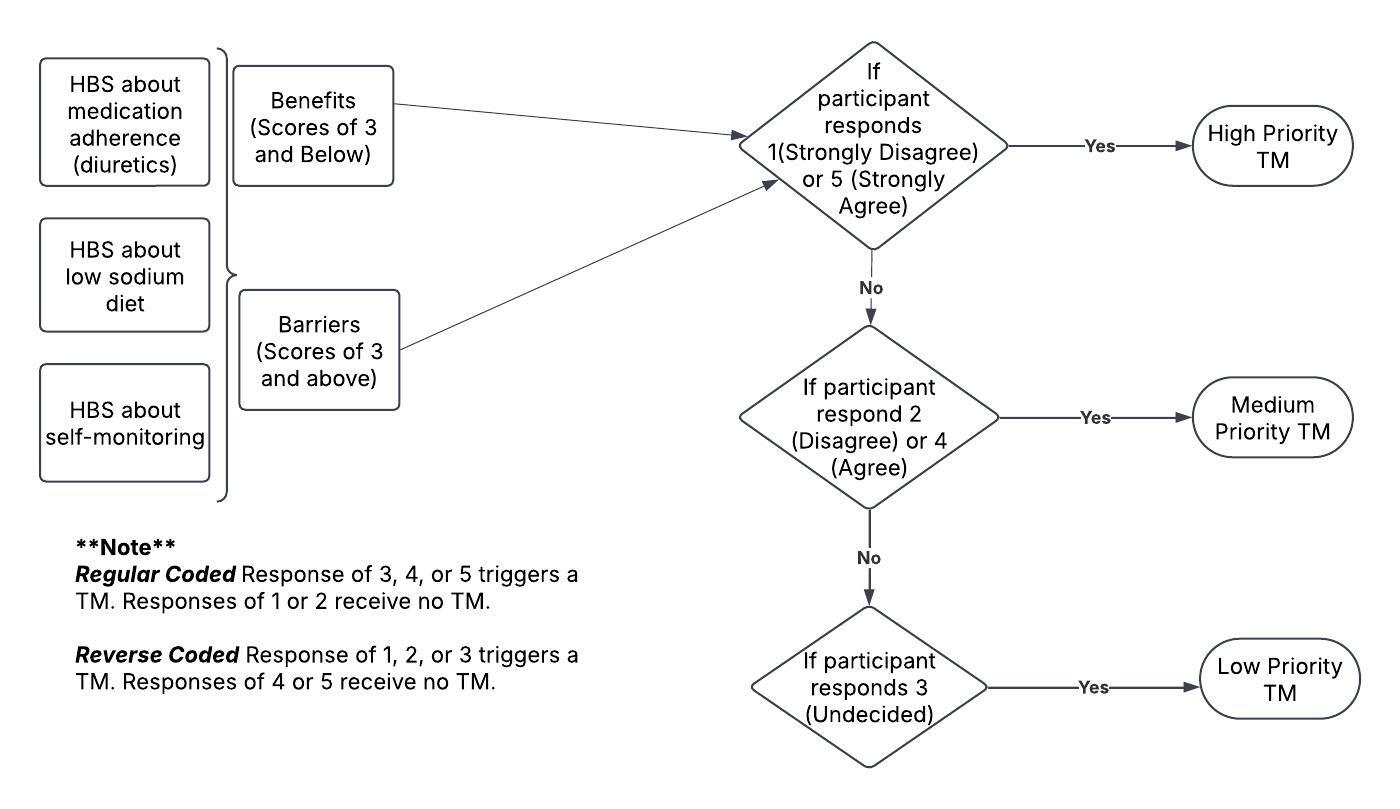


**Figure 1: TM Prioritization Health Beliefs Scales**

***Triggering***: The ARMS is a 4-point scale, answers to regularly coded items trigger a TM for responses of ≥2. Reverse coded items trigger messages for answers ≤3. Responses of 1(regular coded) or 4 (reverse coded) do not receive TM.

| ARMS TM Triggering (4 Point Likert Scale) | | | | |
| --- | --- | --- | --- | --- |
|  | None | Some | Most | All |
| Regular Coded | 1 | 2[TM] | 3[TM] | 4[TM] |
| Reverse Coded | 1[TM] | 2[TM] | 3[TM] | 4 |

**Table 2: ARMS TM Triggering based on participant responses**

***Prioritization***: TM are prioritized by response to each item 4 (regular coded) and 1 (reverse coded) are the highest priority response, 3 (regular coded) and 2 (reverse coded) are the medium priority response, 2 (regularly) and 3 (reverse coded) are the lowest priority.

**
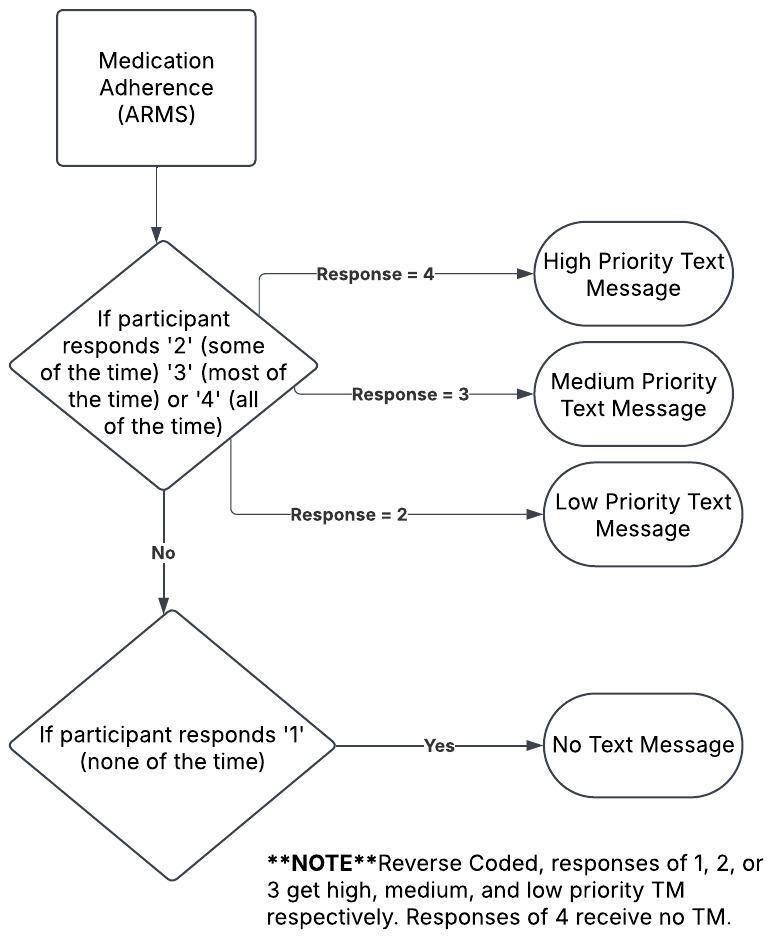
**

**Figure 2: TM Prioritization ARMS**

***Triggering***: The SDBHF is a 4-point scale answers to regularly coded items trigger a TM for responses of ≤3. Reverse coded items trigger messages for answers ≥2. Responses of 4 (regular coded) or 1 (reverse coded) do not receive TM.

| SDBHF TM Triggering (4 Point Likert Scale) | | | | |
| --- | --- | --- | --- | --- |
|  | Never | Sometimes | Frequently | Always |
| Regular Coded | 1[TM] | 2[TM] | 3[TM] | 4 |
| Reverse Coded | 1 | 2[TM] | 3[TM] | 4[TM] |

**Table 3: SDBHF TM Triggering based on participant responses**

***Prioritization***: TM are prioritized based on each participant response to each item as follows: 1 (regular coded) and 4 (reverse coded) are the highest priority response, 2 (regular coded) and 3 (reverse coded) are the medium priority response, 3 (regularly) and 2 (reverse coded) are the lowest priority.


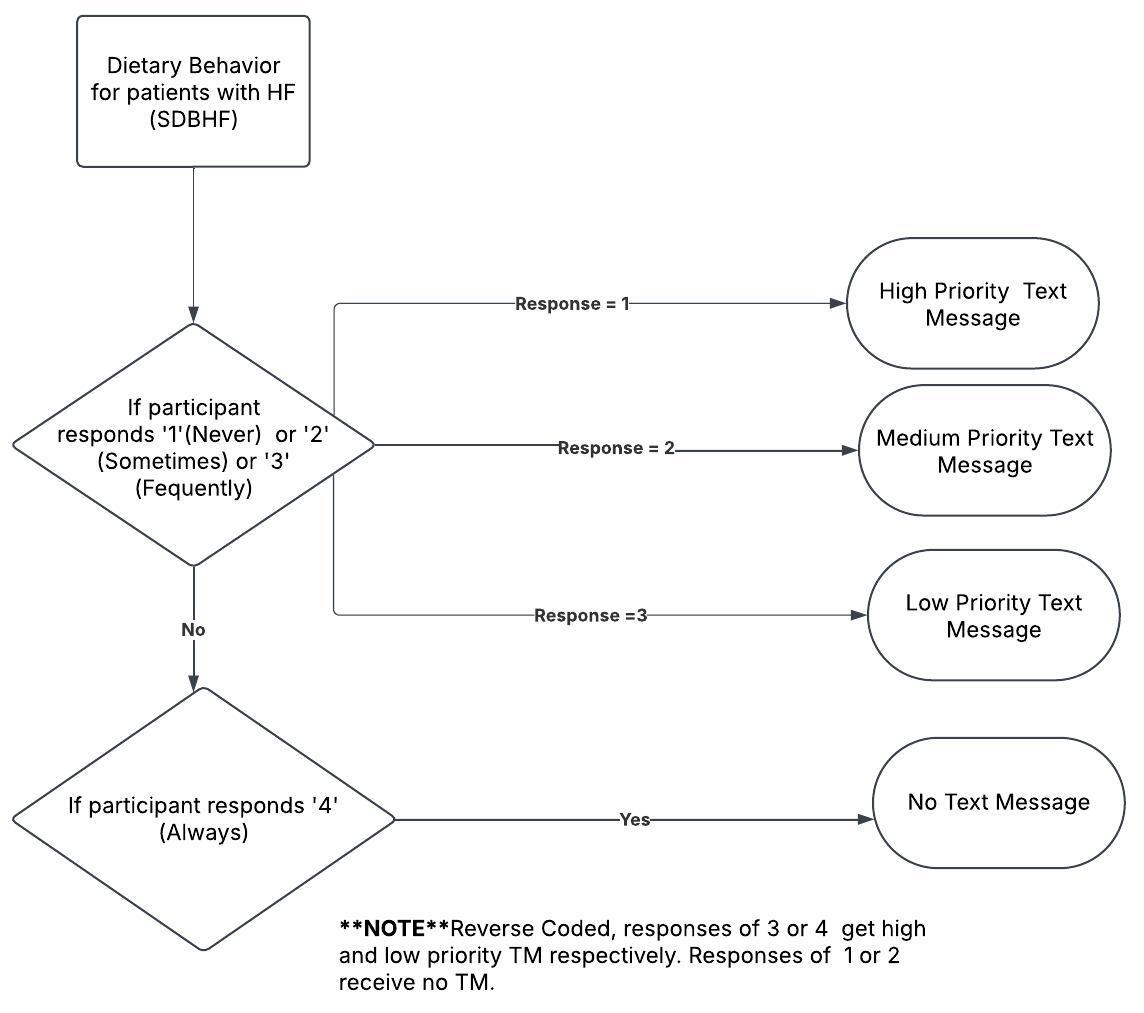


**Figure 3: TM Prioritization SDBHF**

***Sample Messages***

| Target Behavior | Example Text Messages |
| --- | --- |
| Medication Adherence | Take your medication at the same time as an activity you do every day such as cooking, brushing your teeth, or watching your favorite tv show.  It is important to take all of your medicines at the prescribed doses even if you are feeling better. If you feel better every day, it may be because of your medication. Keep taking it and Keep up the good work!  People do sometimes worry about their health. One thing that might help you worry less is to take your medicines as the doctor or nurse tells you. |
| Heart Healthy Diet | Eat with a purpose and listen to your body when you are comfortably full, you do not need to eat everything on the plate.  Make a fist, this is about 1 cup. You can use this when eating out or shopping to measure your portion size.  Before eating out at a restaurant check the menu beforehand and pick out the healthy choices. Focus on baked, steamed, or grilled items. Try to limit or not eat fried food. |
| Symptoms Monitoring | Swelling is a sign that fluid is building up in your body. Check your feet, legs, hands, and abdomen every day for signs of swelling and respond early.  Set aside a few minutes every day to weigh yourself. It helps you find out if there is too much fluid in your body.  Tell your doctor or nurse if you gain more than 3 to 5 pounds since your last doctor’s visit. Your doctor or your nurse may need to adjust your medicines. |
